# Hospital Night Nursing; Anxious to Enjoy a Modern Improved System

**Published:** 1919-02-08

**Authors:** 


*To the Editor of* The Hospital.

### Hospital Night Nursing; Anxious to Enjoy a Modern Improved System.

*To the Editor of* The Hospital.

Sir,—Keenly interested in the contribution of "Modern Experience" for the "other side" of the long-needed discussion on hospital night nursing, I would yet like to enquire the location of the hospital where that early bread-and-butter meal is only the preliminary to a better breakfast "making for great variety" a little later on. I would like to go there when I am ill, for I feel sure it must be a bright exception to a very drab rule. I append the names of several hospitals (in England) where I have personal knowledge, or direct information, that the later lunch consists of a bare cup of milk or cocoa.

Hardly second, in fact, to the night-nursing troubles many would say was the "ordinary diet" of the hospital patient. Please, having begun, do "carry on" until fair chance to sleep and good quality food, well cooked and served, are regarded as the right of every patient, however poor.

As yet, I fear me, they are not common joys.

Pro Bono.

